# AMR mechanisms in *L. interrogans* serovars: a comprehensive study

**DOI:** 10.3389/fcimb.2024.1384427

**Published:** 2024-04-11

**Authors:** Pavlo Petakh, Oleksandr Kamyshnyi

**Affiliations:** ^1^ Department of Biochemistry and Pharmacology, Uzhhorod National University, Uzhhorod, Ukraine; ^2^ Department of Microbiology, Virology, and Immunology, I. Horbachevsky Ternopil National Medical University, Ternopil, Ukraine

**Keywords:** *Leptospira interrogans*, antibiotic, resistance, leptospiral serovars, leptospirosis

## Abstract

Antimicrobial resistance (AMR) is one of the global health challenges of the 21st century. Data regarding AMR mechanisms in *Leptospira interrogans*, the causative agents of leptospirosis, have been relatively limited. Therefore, our study aimed to identify resistance genes and explore potential resistance mechanisms specific to particular serovars. We conducted a comprehensive analysis of 98 Leptospira strains, representing 10 common serovars, using whole-genome sequencing (WGS) FASTA files. Employing the PATRIC tool from the Bacterial and Viral Bioinformatics Resource Center (BV-BRC), we scrutinized the genomes for AMR genes. Our investigation revealed 32 genes associated with AMR, with 20 key genes consistently prevalent across most strains. Notably, we identified unique efflux pump systems in serovar Pomona, indicating distinctive resistance mechanisms in this serovar. In summary, our findings shed light on the genetic landscape of AMR in Leptospira, uncovering both common and serovar-specific resistance elements. The presence of unique efflux pump systems in serovar Pomona introduces a novel dimension to our understanding of resistance mechanisms. These insights underscore the importance of tailored intervention strategies and collaborative efforts between human and veterinary healthcare professionals, as well as environmental scientists, to address the complex dynamics of leptospirosis and its implications for antibiotic resistance.

## 1 Introduction

Antimicrobial resistance (AMR) presents significant challenges in addressing bacterial infections across both animal and human populations (Arnold et al., 2016; Furness et al., 2017). The emergence and dissemination of antimicrobial-resistant microbes over the past few decades pose a severe global health threat (Pulingam et al., 2022). AMR not only increase morbidity and mortality but also substantially impacts treatment duration and costs (Murray et al., 2022).

Although leptospirosis is endemic in tropical geographic areas, it is known as one of the most considerable global zoonotic bacterial infections, which may lead to establishing epidemics on large scales as a result of flooding and strong rainfall (Haake and Levett, 2015; Rajapakse, 2022). The zoonotic infection of leptospirosis annually may cause one million cases with a mortality rate of 6.86% (approximately 60,000 deaths) around the world (Adler and de la Peña Moctezuma, 2010; Petakh and Nykyforuk, 2022; Petakh et al., 2023; Petakh et al., 2022a; Petakh et al., 2022b; Petakh et al., 2022c). The genus Leptospira comprises over 20 species based on DNA relatedness, with more than 350 serovars identified based on surface agglutinating lipopolysaccharide antigens (Adler and de la Peña Moctezuma, 2010; Fouts et al., 2016). These species are broadly categorized into three groups. Saprophytic species like *Leptospira biflexa* are not associated with disease. Pathogenic species such as *Leptospira interrogans* and *Leptospira borgpetersenii* cause leptospirosis globally, ranging from mild or asymptomatic infection to severe forms resulting in multiple organ failure and death. An intermediate group, including *Leptospira fainei* and *Leptospira licerasiae*, may be associated with infection and mild disease (Fouts et al., 2016).

*Leptospira* spp. exhibit intrinsic resistance to various antimicrobial agents, though the specific mechanisms responsible remain unidentified (Adler et al., 1986; Vinod Kumar et al., 2016; Petakh et al., 2024a; Petakh et al., 2024b). Nevertheless, resistance to sulfonamides, neomycin, actidione, polymyxin, nalidixic acid, vancomycin, and rifampicin has facilitated the development of selective media for isolating leptospires (Schönberg, 1981).

In recent research, genes linked to AMR have been explored using the Comprehensive Antibiotic Resistance Database (CARD) definition (McArthur et al., 2013; MaChado et al., 2022). The CARD definition outlines different modes of AMR action:

1. Antibiotic target in susceptible species: genes associated with antibiotic-sensitive wild-type bacterial components may undergo mutations, rendering them not susceptible.
2. Protein altering cell wall charge conferring antibiotic resistance: Genes involved in cell wall alteration, leading to changes in charge that confer resistance to antibiotics.
3. Gene conferring resistance via absence: deletion of specific genes or gene products results in resistance. For instance, deletion of a porin gene can block the entry of drugs into the cell.
4. Antibiotic inactivation enzyme: genes encoding enzymes that catalyze the inactivation of antibiotics, resulting in resistance: inactivation includes chemical modification, destruction, etc.
5. Efflux pump conferring antibiotic resistance: subunits of efflux proteins that pump antibiotics out of a cell to confer resistance.
6. Antibiotic target protection protein: these proteins confer antibiotic resistance by binding the antibiotic target to prevent antibiotic binding.
7. Antibiotic target modifying enzyme: enzymes that confer resistance by modifying (mutational alteration or enzymatic modification) antibiotic targets.
8. Regulator modulating expression of antibiotic resistance genes: mechanism activated by the presence of a specific antibiotic.

Current recommendations for treating human leptospirosis involve penicillin, ampicillin, ceftriaxone, or cefotaxime (Adler and de la Peña Moctezuma, 2010; Haake and Levett, 2015). Alternatives, particularly for those with allergies or in non-hospital settings, include oral doxycycline or azithromycin. In veterinary settings, a penicillin-streptomycin combination is the preferred therapy for acute leptospirosis, although ampicillin, amoxicillin, tetracyclines, tulathromycin, and third-generation cephalosporins have also been utilized (Ellis, 2015). Tilmicosin presents an additional alternative (Alt et al., 2001).

The apparent absence of significant antimicrobial resistance emergence in Leptospira prompts the question of why this has not occurred (Liegeon et al., 2018). Leptospiral infections are typically monomicrobial, limiting opportunities for horizontal resistance gene acquisition. Moreover, there is no experimental evidence of foreign DNA uptake by *Leptospira* spp., although genomic analyses support this notion. Finally, human leptospirosis is a dead-end infection, with human-to-human transmission being extremely rare (Trott et al., 2018).

The study of antimicrobial resistance (AMR) mechanisms in *Leptospira interrogans* serovars is crucial due to the increasing global burden of leptospirosis, a zoonotic disease caused by this bacterium (Boss et al., 2019). Understanding the mechanisms that contribute to AMR in different serovars is essential for developing effective treatment strategies and controlling the spread of resistant strains. In this comprehensive study, we aim to investigate the genetic and phenotypic characteristics of AMR in *L. interrogans* serovars, shedding light on the factors that drive resistance and providing valuable insights for future therapeutic interventions.

## 2 Materials and methods

We conducted an analysis of FASTA files obtained from the whole-genome sequencing (WGS) of 98 strains, representing 10 common serovars in humans and animals (Oliveira et al., 2017). The distribution of strains among the serovars is as follows: Australis (n = 1); Autumnalis (n = 1); Bataviae (n = 6); Canicola (n = 11); Copenhageni (n = 30); Grippotyphosa (n = 6); Icterohaemorrhagiae (n = 11); Manilae (n = 5); Pomona (n = 16); Pyrogenes (n = 11) (**Supplementary Materials**). Strains were collected during 1958 – 2020 years.

We employed the PATRIC tool from the Bacterial and Viral Bioinformatics Resource Center (BV-BRC) to analyze the genomes for antimicrobial resistance (AMR) genes. The Genome Annotation Service in PATRIC uses the k-mer-based AMR gene detection method, which utilizes PATRIC’s curated collection of representative AMR gene sequence variants and assigns to each AMR gene a functional annotation and a broad mechanism of antibiotic resistance (Wattam et al., 2017).

## 3 Results

**Figure 1:**
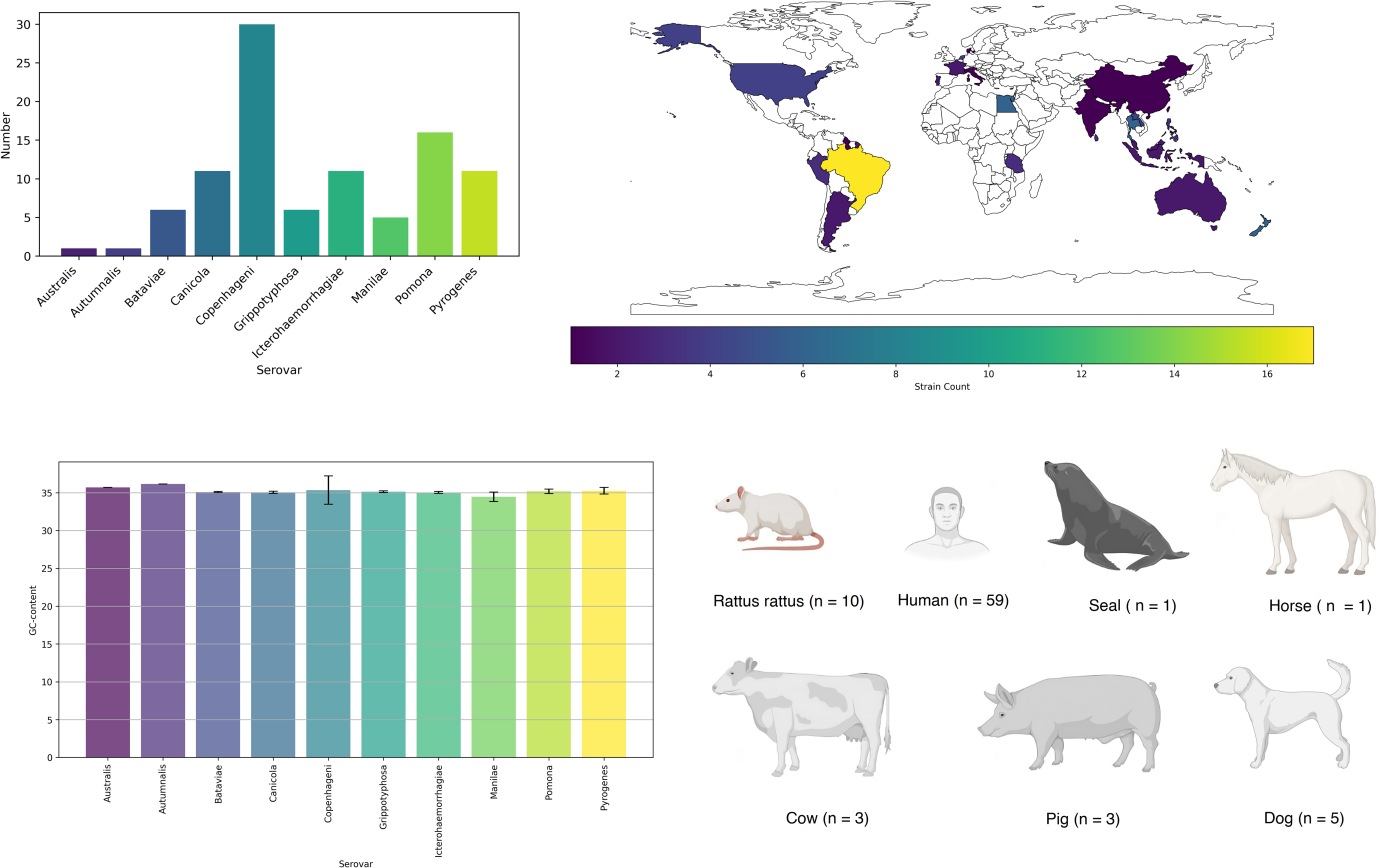
Serovar, isolation country, host distribution, and G/C content of investigated genomes.

**Figure 2:**
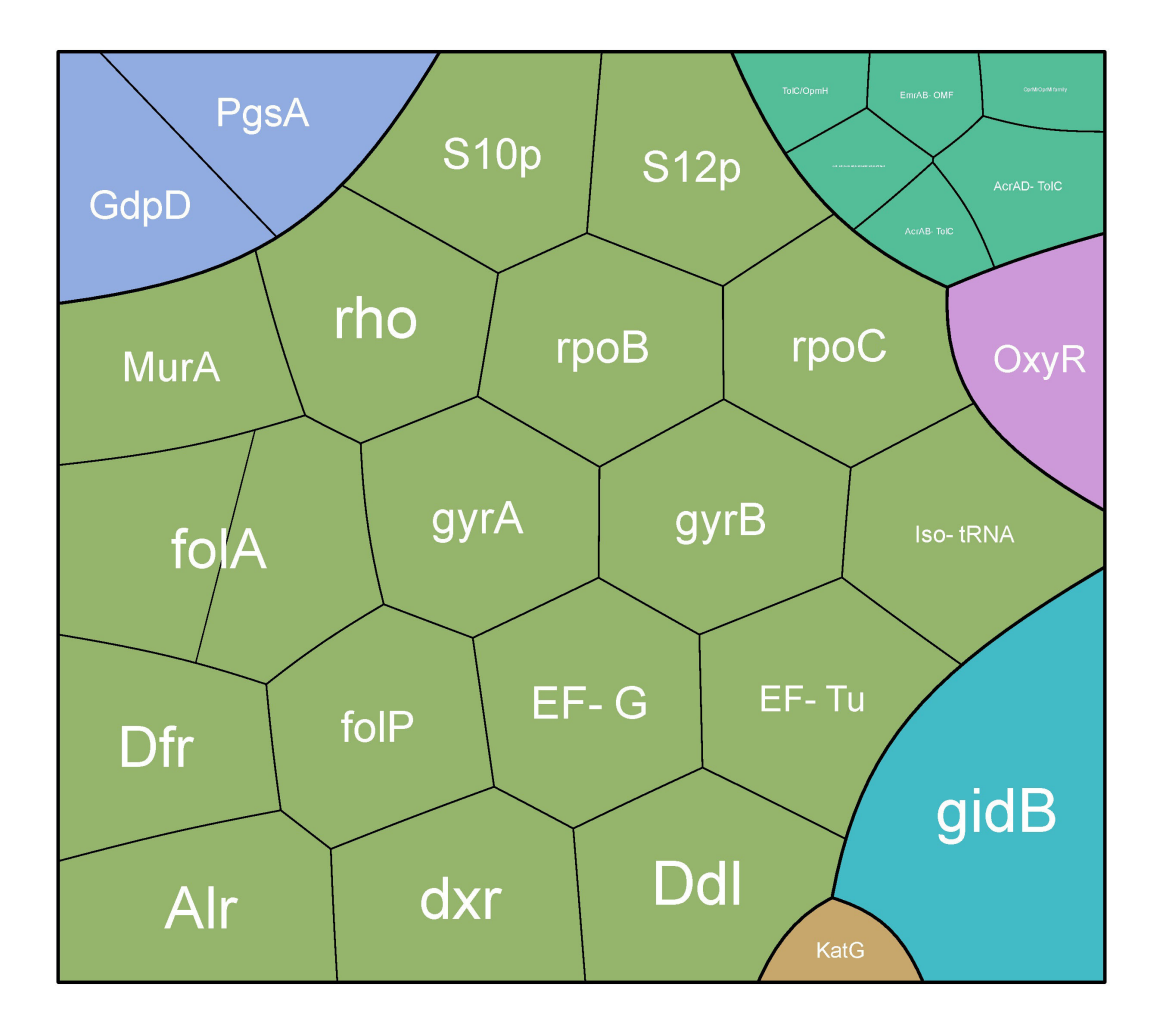
Distribution of AMR genes across all strains.

The majority of strains were isolated in Brazil (n = 17) and the United Kingdom (n = 16) (**Figure 1**). Most of the strains were obtained from humans (n = 59). In general, five main mechanisms of resistance to antibiotics were identified: gene conferring resistance via absence; protein altering cell wall charge conferring antibiotic resistance; antibiotic activation enzyme; regulator modulating expression of antibiotic resistance genes; efflux pump conferring antibiotic resistance (**Figure 2**).

The three predominant and widespread mechanisms were antibiotic target in susceptible species, gene conferring resistance via absence, and protein altering cell wall charge conferring antibiotic resistance, all of which were detected in all studied strains.

Seven genes (*alr*, *ddl*, *dxr*, *EF-G*, *EF-Tu*, *folA*, *dfr*) were observed in all studied strains. These genes are part of the antibiotic target in susceptible species. Antibiotic-sensitive wild-type bacterial components might undergo mutations that render them non-susceptible. All serovars had the *folP* gene, which encodes dihydropteroate synthase, the target enzyme of sulfonamides, except for one strain of serovar Pyrogenes (*Leptospira interrogans* serovar Pyrogenes str. R168). Additionally, *Leptospira interrogans* serovar Pyrogenes str. 200701872 did not have the *gyrB* gene, which encodes the B subunit of the DNA gyrase. *Leptospira interrogans* serovar Copenhageni str. M20 and *Leptospira interrogans* serovar Pyrogenes str. R168 did not have the *MurA* gene, a key enzyme involved in bacterial cell wall peptidoglycan synthesis and a target for the antimicrobial agent fosfomycin. *Leptospira interrogans* serovar Canicola strain Tande did not have the *rpoB* gene encoding the beta subunit of RNA polymerase (*rpoB*), the *rpoC* gene encoding DNA-directed RNA polymerase subunit beta, and the S12p gene encoding the small ribosomal protein.

**Figure 3:**
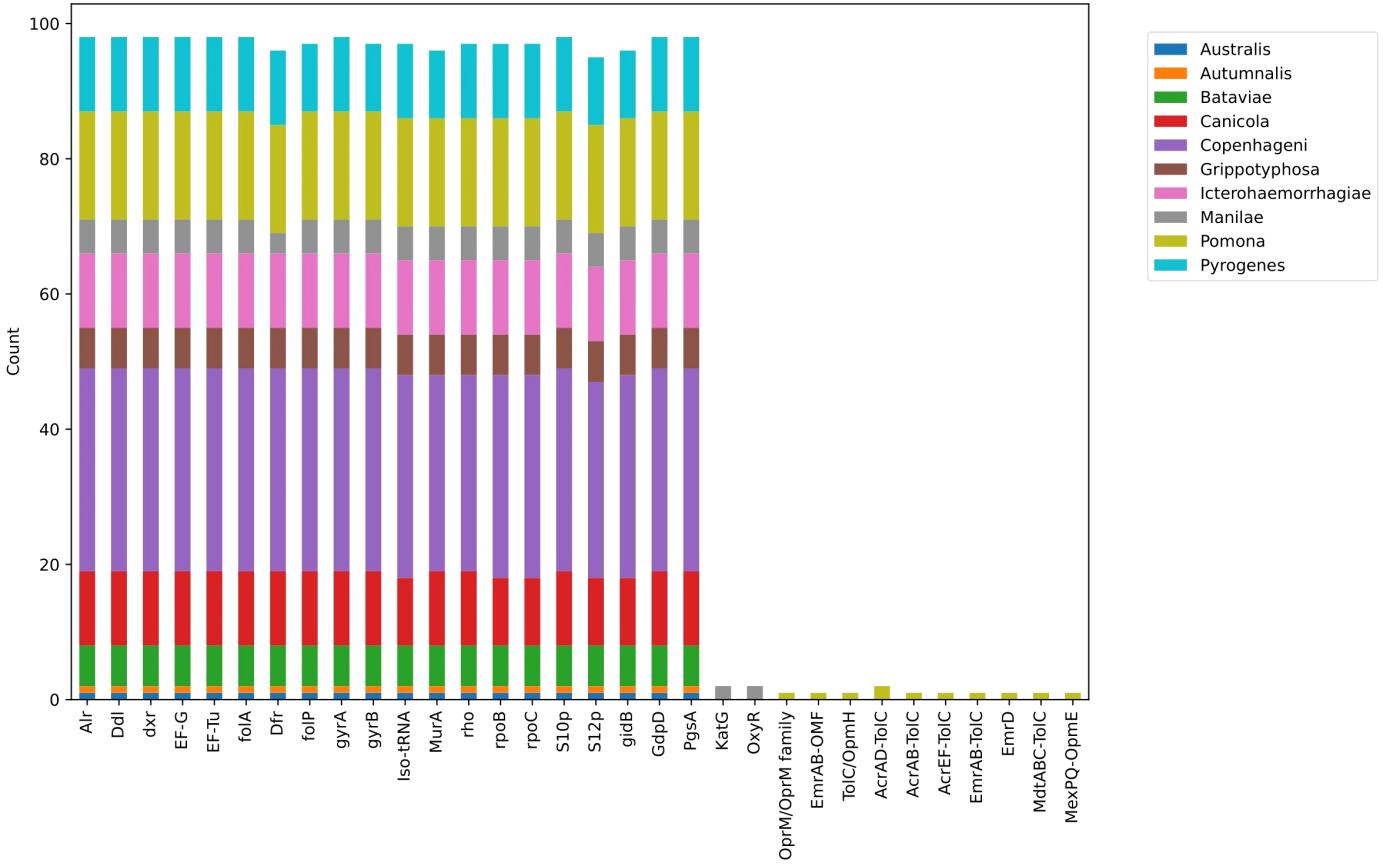
Distribution of AMR genes across all serovars.

Two mechanisms, antibiotic activation enzyme and regulator modulating expression of antibiotic resistance genes, were found among two strains of serovar Manilae. Specifically, the catalase-peroxidase enzyme KatG, encoded by the *katG* gene, and *oxyR*, the canonical orchestrator of the H_2_O_2_ detoxification response, were identified. Additionally, four strains of serovar Pomona exhibited efflux pump conferring antibiotic resistance (**Figure 3**). Specifically, in *Leptospira interrogans* serovar Pomona strain EMY7780, OprM/OprM family and TolC/OpmH were identified. In *Leptospira interrogans* serovar Pomona strain ESR8, AcrAB-TolC, AcrAD-TolC, and TolC/OpmH were observed. Strain P5661 of *Leptospira interrogans* serovar Pomona showed AcrAD-TolC, AcrEF-TolC, EmrAB-TolC, EmrD, MdtABC-TolC, and MexPQ-OpmE. Lastly, in *Leptospira interrogans* serovar Pomona strain Pom_str68, EmrAB-OMF was found.

## 4 Discussion

**Figure 4:**
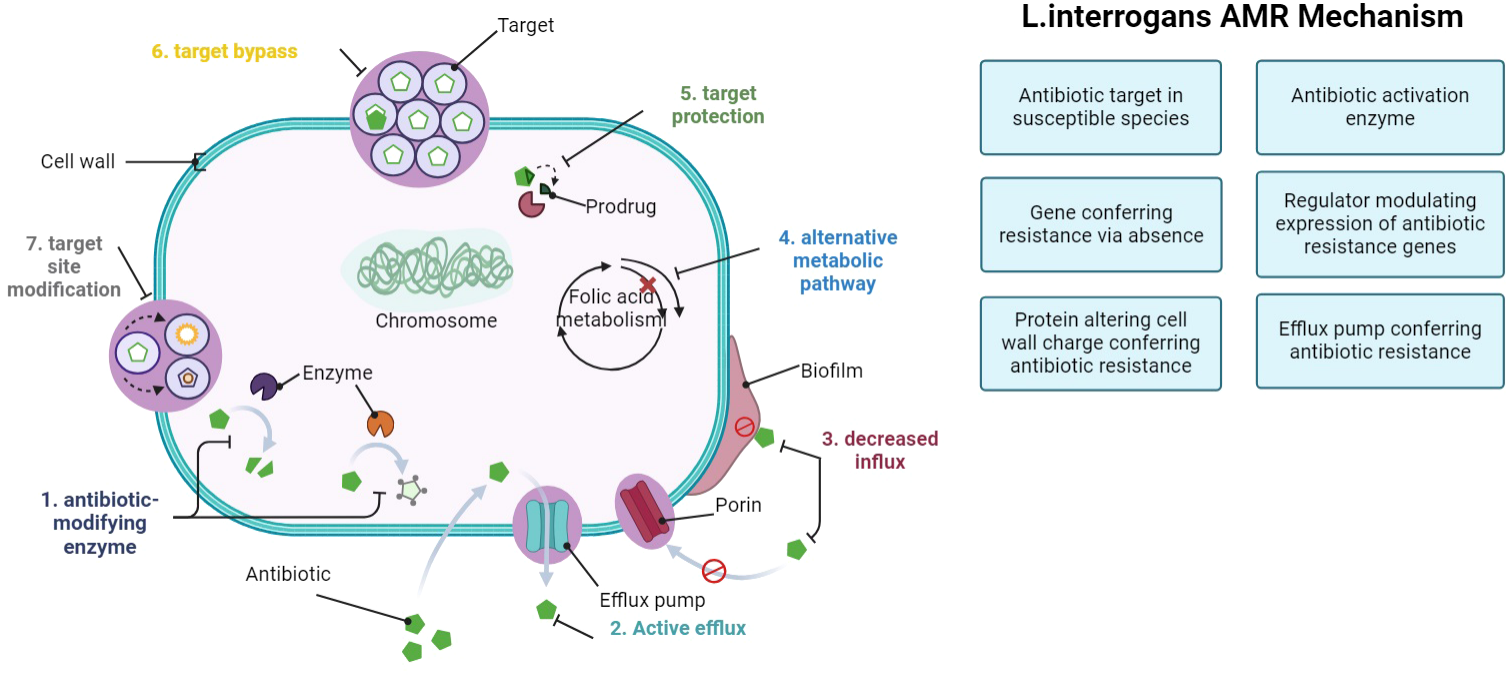
Mechanisms of AMR in *L. interrogans* strains.

In our study of 98 types of Leptospira bacteria, we discovered 32 genes linked to antimicrobial resistance (AMR). Out of these, 20 key genes consistently stood out in most strains. The highest number of genes was associated with a mechanism that makes antibiotics less effective in susceptible species, involving 17 different genes (**Figure 4**).

Some crucial genes, such as *alr* and *ddl*, encode D-alanine racemase (Alr) and D-alanine:D-alanine ligase (Ddl), which are peptidoglycan biosynthetic enzymes. Feng reported that the overproduction of Alr in Mycobacterium smegmatis determines a d-Cycloserine resistance phenotype (Feng and Barletta, 2003).

Another important gene is *murA*, which is a key enzyme involved in bacterial cell wall peptidoglycan synthesis and is a target for the antimicrobial agent fosfomycin, a structural analog of the MurA substrate phosphoenol pyruvate (Kumar et al., 2009).

The *dxr* gene plays a significant role in building the cell membranes of bacteria. Fosmidomycin is a phosphonic antibiotic that inhibits 1-deoxy-D-xylulose 5-phosphate reductoisomerase (Dxr), the first committed step of the non-mevalonate pathway of isoprenoid biosynthesis. In Mycobacterium tuberculosis, Dxr is encoded by Rv2870c, and although the antibiotic has been shown to inhibit the recombinant enzyme, mycobacteria are intrinsically resistant to fosmidomycin at the whole-cell level (Brown and Parish, 2008).

For DNA replication and repair, DNA gyrase subunits (*gyrA* and *gyrB*) are like the repair crew for the bacterial DNA (Chien et al., 2016). Efflux pumps and mutations in *gyrA*, associated with fluoroquinolone resistance, have been induced *in vitro* in *Brucella* spp. strains. Think of these as defense mechanisms that bacteria develop to resist certain antibiotics.

An interesting discovery was made regarding certain strains of serovar Pomona. These strains possess different efflux pump systems that expel drugs, impacting bacterial resistance. This emphasizes the need to understand these variations for effective treatment. In study *L. interrogans* serovar Pomona isolates from swine in Brazil were found to be resistant to fluoroquinolones (Miraglia et al., 2008). We think that overexpression of efflux pumps might be the reason.

In a separate study by Antara Chakraborty and colleagues, 46 Leptospira isolates from rats were tested for their susceptibility to 14 antimicrobial agents (Chakraborty et al., 2010). All of the strains were found to be sensitive to ampicillin, cefotaxime, ciprofloxacin, norfloxacin, doxycycline, erythromycin, and streptomycin. In contrast, the tested isolates showed resistance to amphotericin B, 5-fluorouracil, fosfomycin, trimethoprim, sulfamethoxazole, neomycin, and vancomycin.

## 5 Conclusions

In the end, our research on AMR mechanisms in *Leptospira interrogans* serovars found 32 genes linked to resistance, with 20 key genes present in all strains. We believe these genes form the basis that ensures leptospira’s resistance to certain types of antibiotics. The diverse resistance profiles observed among different *Leptospira interrogans* serovars underscore the complexity of antimicrobial resistance mechanisms in this bacterium.

The identified serovar-specific resistance mechanisms raise the prospect of tailoring therapeutic approaches based on the prevalent serovar in a specific geographical area. A nuanced understanding of the resistance patterns associated with each serovar could lead to more effective and targeted treatment strategies.

Efflux pump systems identified in serovar Pomona, introduce a dimension of complexity to the management of leptospirosis. The potential contribution of these systems to AMR highlights the need for future research.

Given its status as a zoonotic disease, understanding the interplay between human, animal, and environmental factors is crucial for comprehensive intervention strategies. The identified resistance mechanisms should prompt collaborative efforts between human and veterinary healthcare professionals, as well as environmental scientists, to address the multifaceted nature of leptospirosis and its potential impact on antibiotic resistance dynamics.

## Data availability statement

The raw data supporting the conclusions of this article will be made available by the authors, without undue reservation.

## Author contributions

PP: Conceptualization, Investigation, Visualization, Writing – original draft. OK: Conceptualization, Supervision, Visualization, Writing – original draft, Writing – review & editing.
